# A Systematic Review of Atypical Teratoid Rhabdoid Tumor in Adults

**DOI:** 10.3389/fonc.2018.00567

**Published:** 2018-11-28

**Authors:** Vivien Chan, Alessandro Marro, J. Max Findlay, Laura M. Schmitt, Sumit Das

**Affiliations:** ^1^Division of Neurosurgery, University of Alberta Hospital, Edmonton, AB, Canada; ^2^Department of Radiology, University of Toronto, Toronto, ON, Canada; ^3^Division of Neuropathology, University of Alberta Hospital, Edmonton, AB, Canada; ^4^Neuroscience and Mental Health Institute, University of Alberta, Edmonton, AB, Canada

**Keywords:** Atypical teratoid rhabdoid tumor, rhabdoid tumor, adult, systematic review, CNS tumor, brain tumor

## Abstract

**Background:** Atypical teratoid/rhabdoid tumor in adults is a relatively rare malignant neoplasm. It is characterized by the presence of rhabdoid cells in combination with loss of either the INI1 or BRG1protein from the tumor cells.

**Methods:** A systematic review was conducted using MEDLINE using the terms “atypical teratoid rhabdoid tumor” AND “adult.” The systematic review was supplemented with relevant articles from the references. Cases were included if the pathology was confirmed by loss of INI1 or BRG1. We included a case from our institution. The dataset was analyzed using descriptive statistics and log-rank test.

**Results:** A total of 50 cases from 29 articles were included in this study. The average age at diagnosis was 36.7 years. The most common locations reported are the sellar region and cerebral hemispheres (without deep gray matter involvement). Of the 50 cases, 14 were reported to show evidence of dissemination. The average overall survival was 20 months. There was a significant difference in survival between the adjuvant therapy groups (*p* = < 0.0001).

**Conclusion:** Atypical teratoid rhabdoid tumor of the central nervous system in adults is a rare neoplasm associated with a poor prognosis in a majority of patients. The treatment and clinical course are highly variable, and it remains unclear which factors impact prognosis.

## Introduction

### Rationale

Atypical teratoid/rhabdoid tumor (AT/RT) of the central nervous system is a highly malignant neoplasm (1). Although this tumor typically affects children younger than 3 years of age, it has been described in adults (1, 2). Clinical presentation can vary depending on the patient's age, location and size of tumor. In adult patients, the most common locations are cerebral hemisphere and sellar region (3). The most characteristic histological feature is the presence of rhabdoid cells; however, this alone is not sufficient for the diagnosis of AT/RT (4). The 2016 WHO Classification of Tumors of the Central Nervous System defined AT/RT by alterations of either INI1 protein (*SMARCB1* gene), or rarely, BRG1 protein (*SMARCA4* gene) (4).

Case reports and case series report an overall poor prognosis, with an average survival of 20 months (5). However, there are cases of adult patients with AT/RT who have survived beyond 17 years from diagnosis (6). Currently, management of AT/RT in adults is based on data extrapolated from pediatric literature (7). Little is known on the optimal management of adult patients with AT/RT (7). Given the limited number of AT/RT cases in adults and the dispersal of these cases in multiple case reports and case series, patient and tumor characteristics, overall prognosis, and impact of extent of resection and adjuvant therapy remains unclear in this patient population. In addition, other previously published systematic reviews and meta-analysis have included tumors that do not have INI1 or BRG1 alterations, resulting in an analysis of a heterogeneous population that may contain tumors that are not molecularly defined as AT/RT (7–14).

### Objectives

This study aimed to systematically review and analyze patient and tumor characteristics, prognosis, and impact of treatment on prognosis in adult patients with AT/RT.

The objective of this study was to report the results of a systematic review of the literature for a pooled analysis of all adult cases of AT/RT confirmed by alterations in INI1 or BRG1. This is the first systematic review to only include cases of AT/RT confirmed with either INI1 or BRG1 alterations.

### Research question

From reviewing all cases of AT/RT in adult patients with confirmed genetic mutation, what are the patient and tumor characteristics and how do extent of resection and adjuvant therapy impact prognosis?

## Methods

### Study design

We report this systematic review in accordance with the Preferred Reporting Items for Systematic Reviews and Meta-Analyses (PRISMA) guidelines.

### Participants, interventions, comparator

We included all articles with cases of AT/RT in adult patients with the neuropathologic diagnosis confirmed by alterations of either *SMARCB1/*INI1 or *SMARCA4/*BRG1 by immunohistochemistry and/or molecular studies. A case of ATRT in an adult patient from our institution was included in the pooled analysis.

### Systematic review protocol

We developed a protocol that had pre-specified objectives, eligibility criteria, data of interest, search strategy, and analyses plan.

### Search strategy

We conducted a systematic review using MEDLINE. We restricted results to articles written in English only. There was no limitation on the date of publication; we reviewed all articles published prior to August 2018. Search terms included “Atypical teratoid rhabdoid tumor” and “adult.” References from relevant articles were used to supplement the systematic review.

### Data sources, studies sections, and data extraction

Duplicate publications were removed. The title and abstracts were reviewed to identify potentially eligible articles. Full texts of potentially eligible articles were obtained and reviewed for eligibility. Data was extracted from eligible articles.

### Data analysis

We collected data on patient demographics, tumor characteristics, survival, and treatment. A case from our institution was included in our analysis. The data was analyzed using descriptive statistics. Log-rank test was used to assess for differences in outcomes between those that received gross total resection, incomplete resection, and biopsy. Log-rank test was used to assess for differences in outcomes between those that received radiotherapy, radiotherapy and chemotherapy, and no adjuvant therapy. Kaplan-Meier curves were used to estimate the survival function.

## Results

### Study selection and characteristics

**Figure 1:**
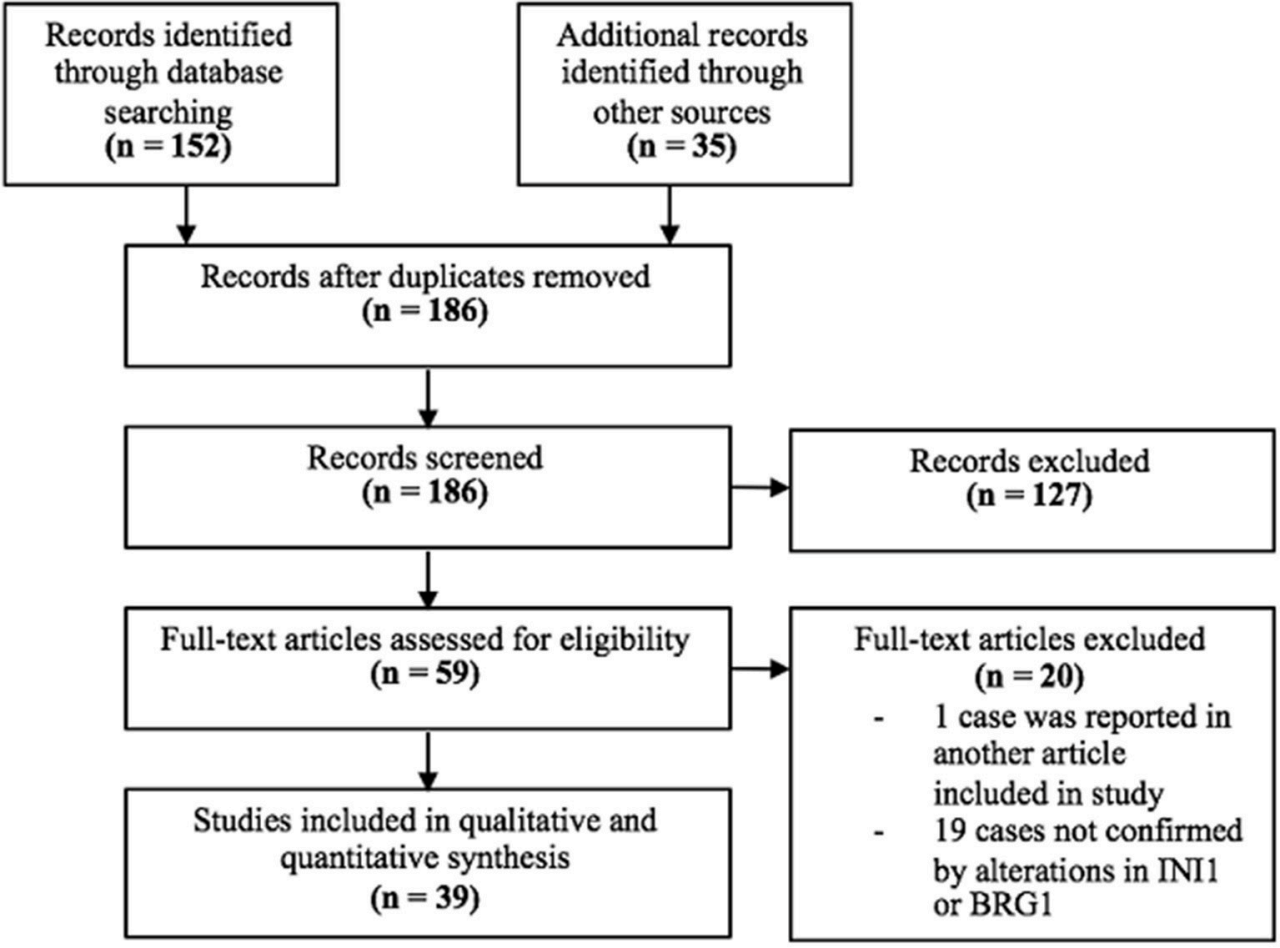
PRISMA Flow Diagram.

A total of 152 results were found from the MEDLINE search (Figure 1). Of the 152 results, 24 articles were deemed relevant to this study. Of these 24 articles, one was removed because the case was previously reported in another article included in this study and 8 were removed because loss of *SMARCB1/*INI1 or *SMARCA4/*BRG*1* was not present or confirmed. The systematic review was supplemented by the references cited in the 24 relevant articles. From the references cited, 35 relevant articles were identified. Of the 35, 11 were removed because loss of *SMARCB1/*INI1or *SMARCA4/*BRG*1* was not present or confirmed. A total of 39 articles were identified to be relevant to this study, 15 from the MEDLINE search and 24 from references cited.

### Synthesized findings

From our systematic review, we found 50 adult patients who were diagnosed with AT/RT from the 39 articles included in this study (Table 1). The Supplementary Table 1 will include all the patients included in the pooled analysis (1, 2, 4–9, 11–42). Of the 50 patients, the average age was 36.69 (*SD* = 15.19) years with range from 18 to 59 years. Of the 39 patients, 34 (68%) were female, 15 (30%) were male, and 1 (2%) was not identified. Signs and symptoms were related to location of the tumor. The most common symptom was headache (*n* = 20, 40%), followed by cranial nerve deficit (*n* = 17, 34%) and visual disturbance (*n* = 14, 28%). Average time with symptoms before surgery was 5.27 (*SD* = 12.72) months. The range was 0–60 months.

**Table 1:** Patient and tumor characteristics from systematic review.

**PATIENT**	
Mean age at diagnosis – year (SD, range)	36.69 (15.19, 18–69)
Female gender – no. (%)	34 (68%)
**TUMOR**	
Location – no. (%)
Spine	3 (6%)
Intracranial	47 (94%)
Hemispheric	16 (32%)
Sellar	23 (46%)
Pineal	3 (6%)
Thalamus	1 (2%)
CP angle	3 (6%)
Cerebellum	1 (2%)
Dissemination – no. (%)	14 (28%)
Composite tumor – no. (%)	6 (12%)
**TREATMENT**	
Surgery – no. (%)
Gross total resection	12 (24%)
Incomplete resection	21 (42%)
Biopsy	1 (2%)
Not reported	16 (32%)
Adjuvant therapy – no. (%)
Radiotherapy and chemotherapy	28 (56%)
Radiotherapy only	8 (16%)
Chemotherapy only	1 (2%)
Stereotactic radiosurgery	2 (4%)
None	4 (8%)
Not reported	7 (14%)
**PROGNOSIS**	
Alive at follow-up – no. (%)	17 (34%)
Mean follow-up – months (SD, Range)	42.53 (48.09, 4–204)
Death – no. (%)	31 (62%)
Mean time to death – months (SD, Range)	20.34 (35.44, 0–168)

*CP angle, cerebellopontine angle*.

Only 3 (6%) were in the spinal cord while the remaining 47 (94%) were intracranial. The most common intracranial location was the sellar region (*n* = 23, 46%), followed by cerebral hemispheres (*n* = 16, 32%). Other reported locations were pineal region (*n* = 3, 6%), cerebellopontine angle (*n* = 3, 6%), cerebellum (*n* = 1, 2%), and, from our case report, thalamus. Over the entire course of the disease, 14 (28%) patients experienced dissemination of disease. 2 (4%) patients were reported to have evidence of dissemination at the time of diagnosis. In 6 (12%) patients, the tumor was a composite tumor involving components of AT/RT and another primary CNS tumor.

Of the 50 cases, 12 (24%) had gross total resection, 21 (42%) had subtotal or partial resection, and1 (2%) had a biopsy. Extent of resection was not reported in 17 cases. With regards to adjuvant therapy, 28 (56%) received combined radiotherapy and chemotherapy, 8 (16%) received radiotherapy only, 1 (2%) received chemotherapy only, 2 (4%) received stereotactic radiosurgery, and 4 (8%) did not receive adjuvant therapy. Of the 36 patients that received radiotherapy, 9 received craniospinal irradiation. Of the 29 patients that receive chemotherapy, 6 received high-dose chemotherapy. Of the 28 patients who received chemotherapy and radiotherapy, 5 patients received both craniospinal irradiation and high-dose chemotherapy. Of those that received adjuvant therapy, 2 patients' death could be attributed to complications and side effects of the therapy.

Of the 50 patients, 31 (62%) had succumbed to their disease with an average time to death of 20.34 (*SD* = 35.44) months. The range was 0 to 168 months. 17 (34%) were alive at last follow-up with a mean follow-up of 42.53 (*SD* = 48.41) months and median follow-up of 28 months (range 4 to 204 months). The range was 4 to 204 months. Of the 17 patients, 11 had no evidence of recurrence at follow-up, with follow-up ranging from 4 to 54 months. Of the 50 patients, 24 patients had reported time to recurrence. The average time to recurrence was 12.4 (*SD* = 16.97) months and median time to recurrence was 5.5 months.

**Figure 2:**
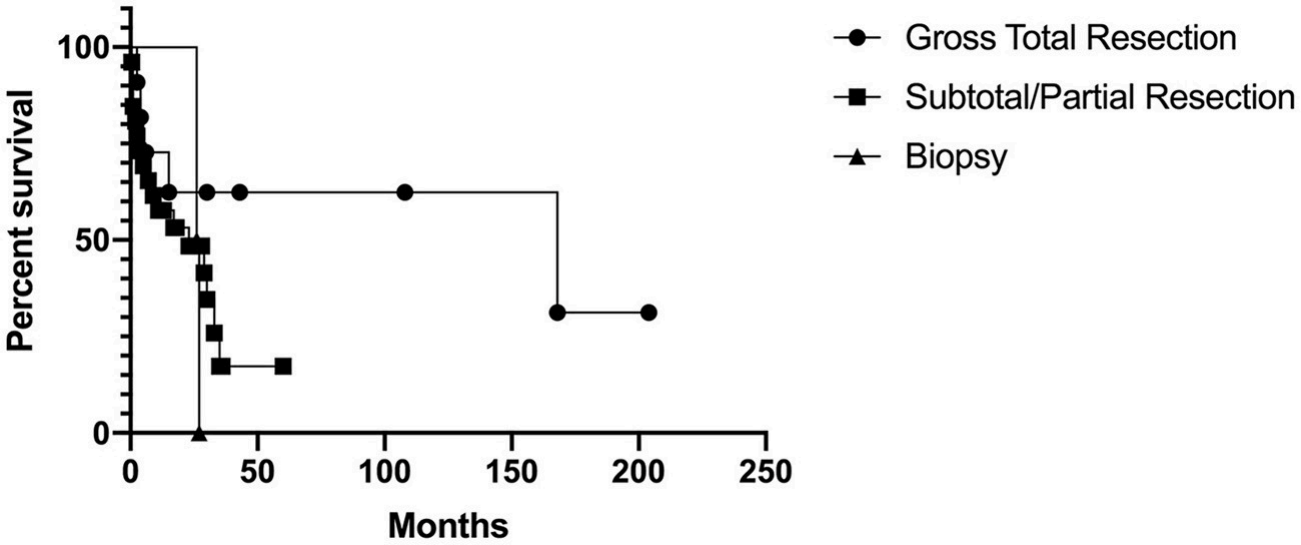
Kaplan-Meier curve for extent of resection.

Of the 12 that had gross total resection, 5 (42%) had passed away with time to death ranging from 3 months to 14 years after surgery. Of the 21 that had incomplete resection, 12 (57%) had passed away with time to death ranging from postoperative to 2.5 years after surgery. The 1 patient that had a biopsy died 2.1 years after diagnosis. When comparing those that received gross total resection, incomplete resection, and biopsy, there was no significant difference on the log-rank test (Chi-square = 3.12, *p* = 0.21). The Kaplan-Meier curve is shown in Figure 2.

**Figure 3:**
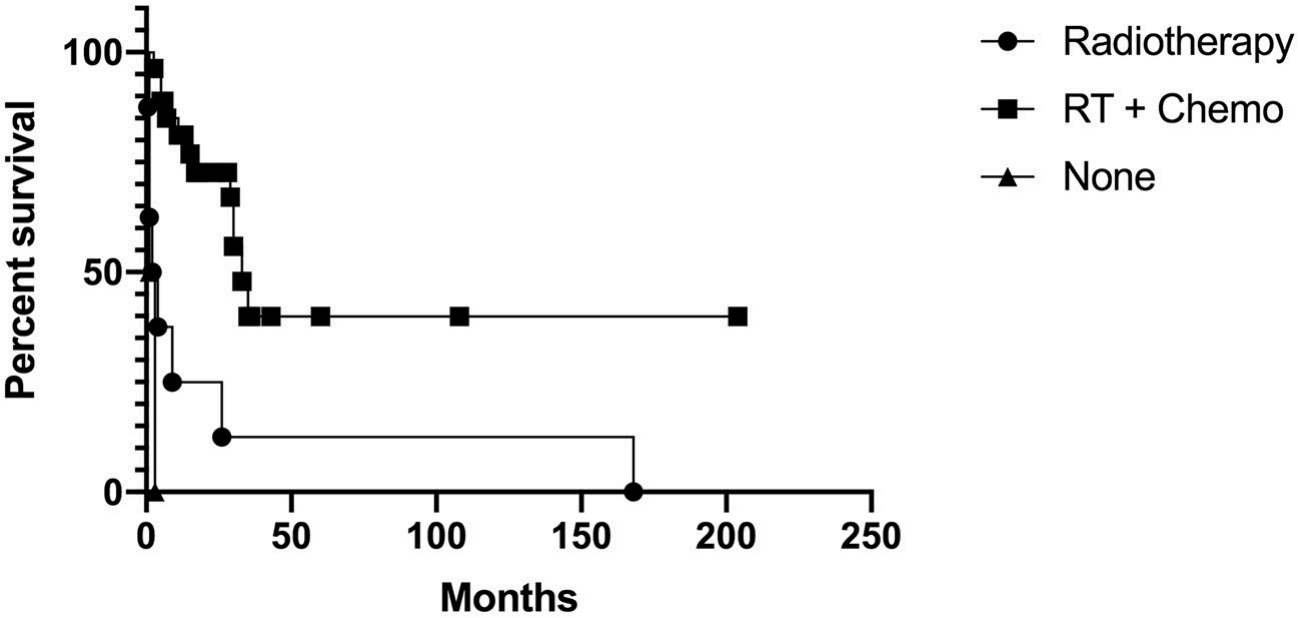
Kaplan-Meier curve for those that received radiotherapy and chemotherapy, radiotherapy only, and no adjuvant therapy.

Of the 28 patients that received combined radiotherapy and chemotherapy, 15 were alive at follow-up, ranging from 6 months to 17 years. Time to death for the remaining 13 of these 28 ranged from 3 months to 3 years after diagnosis. There were no patients alive at follow-up in the radiotherapy only, chemotherapy only, stereotactic radiosurgery only, and no adjuvant therapy groups. The 8 patients treated with radiotherapy died 2 weeks to 14 years after diagnosis. The 1 patient who received chemotherapy died 10 years after diagnosis. The two patients treated with stereotactic radiosurgery died 23 and 27 months after diagnosis. Of the 4 patients who did not receive adjuvant therapy, time to death ranged from the immediate postoperative period to 3 months after surgery. When comparing those that received radiotherapy and chemotherapy, radiotherapy only, and no adjuvant therapy, there was a significant difference in survival (Chi-square = 20.38, *p* = < 0.0001). Patients that received radiotherapy and chemotherapy had a significant increase in survival when compared with patients that received radiotherapy alone (Chi-square = 11.42, *p* = 0.0007) and patients that did not receive adjuvant therapy (Chi-square = 25.71, *p* = < 0.0001). There was no significant difference between The Kaplan-Meier curve is shown in Figure 3.

### Risk of bias

The studies included in this systematic review were case reports and case series. The risk of bias could not be assessed using the Cochrane Collaboration's tool for assessing risk of bias.

## Discussion

### Summary of main findings

In accordance with the 2016 WHO Classification of Tumors of the Central Nervous System, we included only cases with confirmed mutation of INI1. Approximately 20–25% of cases have homozygous deletions of INI1 (43, 44). The majority have a mutation in one allele with a second allele lost due to monosomy 22, deletion of 22q11.2, or an acquired copy number neutral loss heterozygosity (43, 44). Reported mutation hotspots are exons 5 and 9 (5, 43). Our review did not reveal any cases of confirmed BRG1 alteration.

Ostrom et al. reported the most common location for adults to be the cerebral hemisphere, followed by the sellar region (3). In contrast, our review of published cases found the most common location to be the sellar region (46%), followed by cerebral hemisphere (32%). It has previously been suggested that sellar AT/RT represent a different subgroup given its distinct clinicopathological and genetic features (12, 5). Sellar AT/RTs were reported to be found almost exclusively in females and not in the pediatric population (5, 15). Our study found 21 of 23 cases of sellar AT/RT to occur in female patients, suggesting, although much less common, they can occur in males as well. In contrast, male-female ratio for non-sellar AT/RTs in our study were roughly even (46% female, 54% male). Sellar AT/RTs also seem to have a higher prevalence of biallelic *INI1* alterations compared to AT/RT in other locations (5). A recent study, however, argues that sellar AT/RT does not form its own molecular subgroup, but can be clustered into the ATRT-MYC subgroup, one of the three recently described molecular subgroups (16, 45). However, AT/RT in the sellar location appears to have a different mutational spectrum than other tumors in the ATRT-MYC subgroup. Cases of AT/RT concurrent with other central nervous system tumors, such as pleomorphic xanthoastrocytoma (PXA), glioma, ependymoma, and prolactinoma have also been described (13, 17–21). Nobusawa et al. reported a case of a tumor with biphasic appearance consistent with histological features of ependymoma and AT/RT (17). C11 ORF95-RELA fusion, a genetic marker of supratentorial ependymomas, was present in both ependymoma and AT/RT components. However, only the AT/RT component had loss of nuclear expression of *INI1*. Similarly, Chacko et al. reported a composite tumor with features of AT/RT and PXA (18). The PXA component was GFAP positive. Loss of INI1 was seen in the AT/RT component, but retained in the PXA component. It is hypothesized that a postclonal inactivation of *INI1* exists in a subset of the original tumor cells, leading to a composite tumor with features of AT/RT and another central nervous system tumor (17–21). The clinical significance of a concurrent CNS tumor with ATRT is unclear given the paucity of data in the literature.

The propensity for AT/RT to spread through the subarachnoid space is well known (6). In our study, only 2 patients were reported to have evidence of dissemination at the time of diagnosis. However, this value is likely an underestimation since most patients did not receive whole neuraxis imaging at the time of diagnosis. We found 28% of patients had dissemination outside of the original tumor site over the course of their disease. This figure is consistent with previously reported values (46–48). Dissemination is usually within the central nervous system; however, distant metastasis to the lung has been described (22). Even with gross total resection and adjuvant therapy, there are cases of distant metastasis at time of recurrence (19, 23–25). In our pooled analysis, of the 12 cases with gross total resection, we found 4 with dissemination. Although dissemination has been typically associated with poor prognosis, overall outcome still seems to be highly variable with some patients doing well with whole neuraxis radiotherapy and chemotherapy despite presence of dissemination at the time of diagnosis (46). In our study, we found variable outcomes in those that received whole neuraxis radiotherapy, as well as those that received high-dose chemotherapy. In a large majority of the cases that received whole neuraxis radiotherapy or high-dose chemotherapy, these measures were undertaken after discovery of rapid recurrence or new metastatic lesions. Therefore, it is difficult to draw conclusions on the impact of whole neuraxis radiation and high-dose chemotherapy on the course of the disease.

There are clinical differences and similarities between the pediatric population and the adult population. In the pediatric population, there is a higher incidence in males. In a national retrospective study, 62% of reported pediatric cases occurred in males (49). This is contrast to our study which report 32% of the patients were male. In the same study, half of the patients had tumors in the infratentorial location. Whereas, in adult patients the most common locations are sellar and hemispheric. AT/RT carries a poor prognosis in both the adult and pediatric populations. In our study, we report an average survival of 20 months. This is consistent with findings reported in other studies (5, 50, 51). This is comparable to the reported median survival of 13.5 to 16.8 months in the pediatric population (49, 50). In both adult and pediatric populations, the prognosis can be highly variable. In the study by Hilden et al. on pediatric patients with AT/RT, 33% had no evidence of disease at follow-up, with a follow-up range of 9.5–96 months (50). Similarly, in our study 22% had no evidence of disease at last follow-up, with follow-up ranging from 4 to 60 months. Median time to recurrence in the pediatric population was the same as the reported value for adults in this study (49). In the pediatric population, 38% had metastatic disease during the course of their disease (49). This is comparable to the 28% reported in this study. Interestingly, in both pediatric and adult populations metastatic disease was not prognostic.

Radiological findings of AT/RT in adult patients are similar to those reported in pediatric cases (10, 52–55). A study by Kanoto et al. on adult AT/RT found 91% were hyperattenuated on CT, 80% were hypointense on T1-weighted imaging, 72% were mixed-intensity on T2-weighted imaging, 100% had restricted diffusion, 70% had heterogeneous enhancement, 56% had cyst/necrosis, 42% had hemorrhage and 33% had calcifications (56). The high density on CT and diffusion restriction on MRI is suggestive of high cell density (56, 57). The ADC value and CT density are similar to lymphoma, germinoma, and what was previously termed primitive neuroectodermal tumor (58–60).

From our analysis, there was no difference in survival between those that received gross total resection, incomplete resection, and biopsy. Based on our analysis, it remains unclear whether extent of resection impacts survival. Current adjuvant therapy treatment for AT/RT has been extrapolated from pediatric literature, which has shown poor response to adjuvant therapy (7, 47, 48). In our analysis, 15 of 16 patients that were alive at follow-up received chemotherapy and radiation; it was unknown whether the remaining patient received chemotherapy or radiotherapy. There were no patients alive at follow-up in the radiotherapy only, chemotherapy only, stereotactic radiosurgery (SRS) only, and no adjuvant therapy groups. In the no adjuvant therapy group, time to death ranged from postoperative to 3 months. Given the small number of patients in the chemotherapy only and radiosurgery only group and the large range in time to death in the radiotherapy only group (2 weeks to 14 years), it remains unclear how adjuvant therapy impacts survival. In our analysis, we found patients who received chemotherapy and radiotherapy had increased survival. However, survival associated with adjuvant therapy may be impacted by confounding factors. Most of the patients received chemotherapy and radiation unless the patient refused therapy, was too functionally impaired, or the patient succumbed due to rapidly progressive disease. The confounding factor in this context may therefore be the health and/or functional status of patients as only otherwise healthy patients or patients who were less functionally impaired received both chemotherapy and radiation while others may have only received chemotherapy only, radiotherapy only, radiosurgery only, or no adjuvant therapy.

### Limitations

One of the limitations of this study is the limited ability to draw robust conclusions due to the small study population and confounding factors. It is difficult to conclude from this study whether extent of resection or adjuvant therapy provides a real survival benefit or whether there were confounding factors present. Another limitation is the amount of data available in case series and case reports. Although most case series and case reports contain a large amount of specific detail such as patient factors, tumor characteristics, pathological characteristics, treatment, and prognosis, the content of the data is inconsistent from one case series/report to another, which prevented more rigorous statistical analysis, such as regression analysis, and clinically relevant analysis, such as event-free survival, from being done.

## Conclusions

AT/RT in adults is a rare malignant neoplasm of the central nervous system diagnosed based on alterations of either INI1 or BRG1. The prognosis remains poor, with an average survival of less than 2 years. There is however a subset of patients who seem to have a much longer survival after repeat resections and adjuvant therapy. Although the sellar region and cerebral hemispheres tend to be the most common locations for AT/RT in adults, our systematic review demonstrate that AT/RT can also occur in less well known locations. From our systematic review, extent of resection may not be an important factor in prognosis, but adjuvant therapy may have an impact on prognosis. However, it is difficult to draw conclusions given the small population and confounding factors. Case reports and systematic reviews of rare malignant neoplasms remain an important component of literature in oncology as it provides information that may reveal clinicopathological patterns and factors that impact prognosis.

## Data availability statement

All datasets generated for this study are included in the manuscript and the supplementary files.

## Author contributions

VC contributed to design of the study, systematic review for creation of dataset, statistical analysis, and writing the first draft of the manuscript. AM and SD contributed to writing the first draft of the manuscript. LS, JF, and SD contributed to conception of the study. All authors contributed to manuscript revision, read and approved the submitted version.

### Conflict of interest statement

The authors declare that the research was conducted in the absence of any commercial or financial relationships that could be construed as a potential conflict of interest.
