# Supplementary material for: A Systematic Review of Atypical Teratoid Rhabdoid Tumor in Adults
Source: Front Oncol. 2018 Nov 28;8:567. doi: 10.3389/fonc.2018.00567 (PMC6279935; doi:10.3389/fonc.2018.00567)
Supplement: Supplementary file 1 [file Table_1.DOCX]

Supplementary Table. Summary of cases of AT/RT of the central nervous system in adults with *INI1* mutation. CP angle = cerebellopontine angle, GTR = gross total resection, RT = radiotherapy, CS RT = Craniospinal radiotherapy, Chemo = chemotherapy, HD Chemo = high-dose chemothereapy, SRS = stereotactic radiosurgery, PXA = pleomorphic xanthoastrocytoma.

Year of Publication Age (yrs) Gender Tumour Location Dissemination Surgery Adjuvant Therapy Outcome Composite Tumour

Wyatt-Ashmead 2001 18 M Right frontal - - - Died 22 weeks Yes (glioma)

Bruch 2001 34 F Parietal - - - Died 6 months

21 F Spinal cord - - - Died 6 months

Raisanen 2004 45 M R cerebellum - - None -

Raisanen 2005 20 F Sellar - - RT, Chemo 28 months

31 F Sellar Yes - RT Died 9 months

Ingold 2006 45 F Pineal region Yes - RT, Chemo Died 7 months

Chacko 2007 23 M Right frontal - Incomplete RT Died 1 month Yes (PXA)

Zarovnaya 2007 43 F Spinal cord Yes Incomplete RT, Chemo Died 2.5 years

Makuria 2008 25 F Right frontal - GTR RT, Chemo 17 years

42 M Right frontoparietal - Incomplete RT, Chemo 18 months

23 M Left temporal - GTR RT, Chemo 30 months

Arita 2008 56 F Sellar Yes Incomplete SRS Died 23 months

Chi 2009 20 - Supratentorial - Biopsy CS RT Died 2 years

Samaras 2009 18 M R frontotemporal No GTR RT Died 4 months

Takei 2010 33 F Pineal region - Incomplete CS RT, Chemo 13 months

Kleinschmidt 2010 67 F Right occipital - - - Died 36 weeks Yes (glioma)
 -DeMasters

Schneiderhan 2011 57 F Sellar - GTR RT, Chemo 6 months

2011 61 F Sellar - GTR RT, Chemo 9 years

Takahashi 2011 27 F Left parietal - Incomplete None Died 3 months

Kuge 2012 20 F Pineal region - Biopsy SRS Died 27 months

Yamamoto 2012 22 F Right occipital Yes GTR RT, Chemo Died 5 months Yes (glioma)

Roy 2013 24 F Right temporal - Incomplete CS RT, Chemo 11 months

Chou 2013 43 F Sellar Yes - RT Died 2 weeks

Moretti 2013 60 F Sellar Yes Incomplete RT, Chemo Died 30 months

Park 2014 42 F Sellar - Incomplete CS RT, HD Chemo 2 years

Slemp 2014 19 M Right temporal - GTR RT, Chemo 43 months

Shitara 2014 44 F Sellar Yes Incomplete RT, Chemo Died 17 months

Sinha 2015 65 M Spinal cord Yes Incomplete RT Died 1 month

Biswas 2015 48 F Sellar Yes GTR CS RT, Chemo Died 10 weeks

Wang 2015 22 F Left CP angle Yes GTR CS RT, HD Chemo Died 15 months

Lev 2015 36 F Sellar - Incomplete RT, Chemo Died 29 months

Nobusawa 2016 24 M Left frontal - GTR CS RT, HD Chemo 2.5 years Yes (ependymoma)

69 F Sellar No Incomplete RT, Chemo 24 months

Larran 2016 43 F Sellar - Incomplete None Died 5 weeks

Yu 2016 30 M Left CP angle - GTR RT, Chemo -

Almalki 2017 36 F Sellar - Incomplete RT, Chemo 3 years

Horiguchi 2017 24 M Left occipital - Incomplete RT, Chemo 5 years

Nishikawa 2017 42 F Sellar - Incomplete SRS, Chemo Died 11 months

Schweizer 2017 35 M Right frontal Yes GTR RT Died 14 years

Nakata 2017 26 F Sellar - - RT, Chemo Died 33 months

21 F Sellar - - RT, Chemo Died 35 months

Dardis 2017 29 F Left CP angle Yes - CS RT, HD Chemo Died 5 months

Dardis 2017 36 M Sellar Yes Incomplete CS RT, HD Chemo 2.5 years

Johann 2018 48 F Sellar - - - 4 months

66 M Sellar - - - 54 months

20 F Sellar - - HD Chemo Died 120 months

46 F Sellar - - - Died postoperative

Barresi 2018 59 F Sellar No Incomplete RT Died 2 months Yes (prolactinoma)

Our case 2018 27 M Right basal ganglia - Incomplete None Died postoperative
